# Exercise and physical activity for people with chronic kidney disease: A national survey of nephrologist practice patterns in Saudi Arabia

**DOI:** 10.1097/MD.0000000000040394

**Published:** 2024-11-01

**Authors:** Enad Alsolami, Sami Alobaidi

**Affiliations:** a Department of Internal Medicine, University of Jeddah, Jeddah, Saudi Arabia.

**Keywords:** chronic kidney disease, exercise, nephrologist, physical activity, Saudi Arabia

## Abstract

A variety of health benefits are associated with physical activity (PA) in individuals with chronic kidney disease (CKD). The aim of this study was to examine nephrologist practice patterns concerning exercise and PA in CKD patients. This is an online cross-sectional survey study that was conducted between June 2023 and May 2024 in Saudi Arabia. Nephrology fellow, specialists, and consultants in Saudi Arabia formed the study population. A total of 96 physicians participated in this study. Only 9.4% of renal units have exercise programs available to patients. These programs are available mainly for advanced CKD (pre-dialysis) and in-center hemodialysis patients. The major barriers for initiating or expanding exercise programs at their centers are a lack of motivation/interest from front-line staff (55.2%), lack of interest from management (48.3%), and no funding (47.1). Around 56.2% of respondents ask patients about their level of PA. Additionally, 64.6% give patients specific advice on how to increase their level of PA. For the types of exercise most beneficial for people with CKD, 90.6% recommend walking. Around 34.4% of respondents believe that physiotherapists should take ownership in providing exercise counseling and resources to people with CKD. The majority agreed or strongly agreed to recommend exercise in patients with CKD (76.0%). The mean attitude score for the study participants was 32.1 (standard deviation: 3.9) out of 40 (equal to 80.3%); which demonstrates positive attitude towards recommending exercise for CKD patients. Binary logistic regression analysis identified that there is no statistically significant difference between physicians in term of their attitude towards recommending exercise for CKD patients (*P* > .05). This study highlights a critical gap in the availability of exercise programs for CKD patients. Key barriers include lack of interest by staff and management and funding issues. In spite of these barriers, a majority of physicians acknowledge the role of exercise in CKD patients and advice regarding PA on a frequent basis. It suggests recommendations in order to expand the programs, including increasing staff motivation, securing management support, and getting funding for exercise programs and identifying the role of physiotherapists in exercise counseling for CKD patients.

## 1. Introduction

Chronic kidney disease (CKD) is a severe progressive disorder estimated to affect a considerable number of people all over the world, with prevalence varying from 8% to 14%, which is equivalent to about 700 to 840 million individuals globally.^[1–3]^ The CKD incidence is caused by aging, demographic growth, and the rising burden of hypertension, cardiovascular disease, and diabetes, significantly in developed economies countries.^[4]^ The prediction of CKD is also associated with lifestyle factors such as an unhealthy diet, too much salt consumption, alcohol misuse, lack of physical activity (PA), and smoking.^[5]^

An inverse association between PA and physical functioning, quality of life, and mortality has previously been described for patients with CKD.^[6–11]^ Several randomized trials have shown that exercise significantly enhances health-related quality of life, exercise capability, and physical function in patients with CKD.^[8,12,13]^ Accordingly, exercise and PA must be an integral part of the management of patients with CKD.^[14]^ Evidence has also emerged that physician counseling significantly increases patients’ levels of PA,^[15]^ underlying that active support and encouragement of increased PA by nephrologists are needed. According to the “Kidney Disease Outcomes Quality Initiative Clinical Practice Guidelines,” the dialysis and nephrology team should periodically encourage and counsel all dialysis patients to attain higher PA levels.^[16,17]^ Nonetheless, most nephrologists rarely counsel their patients about PA, and among the reasons for this gap in care is a lack of expertise in exercise prescribing and a lack of time.^[18–24]^

In Saudi Arabia, the prevalence of CKD is high and warrants the implementation of proactive interventions.^[25]^ Risk factors for CKD, such as diabetes and hypertension, are projected to increase over the coming decades, thus further compounding the CKD burden.^[25]^ Given that the burden of CKD increases along with the well-established benefits of exercise and PA, there is a need to study these practice patterns to drive health policy decisions and training programs. Still, no prior study in Saudi Arabia has evaluated the practice of nephrologists regarding exercise and PA in CKD patients. Therefore, this national survey aims to examine nephrologist practice patterns concerning exercise and PA in CKD patients.

## 2. Methods

### 2.1. Study design

This is an online cross-sectional survey study that was conducted between June 2023 and May 2024 in Saudi Arabia.

### 2.2. Study population and recruitment procedure

The inclusion criteria for this study were nephrology fellow, specialists, and consultants who are currently practicing their profession in Saudi Arabia. There are no restrictions or limitations associated with gender, age, years of experience, or practice settings. The study excluded healthcare professionals with other specialty areas. In an effort to increase engagement and participation, the survey link was disseminated by the Saudi society of Nephrology. Besides, the survey link was distributed to the target study population using social media platforms (WhatsApp) and colleagues in the medical field.

### 2.3. Sampling procedure

Convenience sampling was implemented to select the sample for the present study. This sampling technique is classified as non-probability sampling that involves selecting participants who are readily accessible and willing to participate in the study.^[26]^ This type of sampling technique is applicable to quantitative research. Physicians who fulfilled the predetermined criteria for inclusion and expressed their willingness to participate in the study comprised the present study. Participants were provided with an informed consent form at beginning of the questionnaire, which enabled them to determine whether to continue their participation in the study or quit. A comprehensive explanation of the research objectives was provided to enhance physicians’ understanding of the significance of their involvement. The criteria for inclusion were comprehensively outlined in the invitation letter for the study.

### 2.4. Questionnaire tool

In this study, the questionnaire tool was adopted from previous study.^[19]^ The questionnaire was administered in English and examined participants’ demographic characteristics (age, gender, country of nephrology fellowship training, number of years practicing nephrology, primary practice setting, and region of nephrology practice), practice settings exercise and PA characteristics, healthcare providers’ practices and opinions on PA for CKD patients, healthcare providers’ confidence, concerns, and perceptions about exercise for CKD patients, and healthcare providers’ attitude towards exercise for CKD patients. Participants’ attitude was examined using 8 questions of 5-point Likert scale format that ranged from strongly disagree to strongly agree. Each response was given a score that ranged from 1 for strongly disagree to 5 for strongly agree. The higher the score the more the positive attitude.

### 2.5. Piloting of the questionnaire tool

Medical professionals affiliated with the University of Jeddah evaluated and validated the questionnaire instrument. The participants were questioned regarding the questions’ clarity, comprehensibility, and face validity, as well as any challenges they encountered in comprehending them. Furthermore, the participants were requested to provide feedback on any inquiries that they found objectionable. Furthermore, a pilot study was conducted to assess the comprehension of the survey instrument among a limited sample of the intended recipients prior to its widespread implementation.

### 2.6. Ethical approval

This study was reviewed and approved by Bioethics Committee of Scientific and Medical Research in University of Jeddah, Jeddah, Saudi Arabia (Reference number: UJ-REC-118). All participants provided their consent before participation in the study.

### 2.7. Statistical analysis

The statistical analysis for this study was performed using the Statistical Package for Social Science Software, version 29. Categorical variables were presented using frequencies and percentages. The normality of continuous variables was examined using histogram, skewness, and kurtosis measures. Continuous variables were presented using the mean and standard deviation (SD). Binary logistic regression analysis was conducted to identify predictors of having positive attitude towards exercise for CKD patients. The dummy variable for the logistic regression model was defined as the mean score of the study participants (which is equal to 32.1). The findings of the regression analysis were presented as odds ratio (95% confidence interval). The significance level was assigned as *P*-value <.05.

## 3. Results

A total of 96 physicians participated in this study. The participants’ mean age was 43.3 years (SD = 8.7), with 69.8% being males. Regarding the nephrology fellowship training, 32.3% completed the Saudi board, 24.0% had a Masters or PhD/fellowship in Egypt, and 15.6% trained through the Royal College of Physicians and Surgeons of Canada or fellowship in Canada. Participants reported a mean of 12.3 years (SD = 9.2) of nephrology practice experience. The majority had primary practice locations in Ministry of Health hospitals (43.8%), with other common locations being non-Ministry of Health hospitals (30.2%), private hospitals (18.8%), and outpatient dialysis units only (14.6%). Geographically, 67.7% of participants were based in the Western region, with others located in the Central (11.5%), Eastern (15.6%), Northern (1.0%), and Southern (4.2%) regions (Table 1).

**Table 1:** Demographic and practice characteristics of the study participants.

Variable	Frequency	Percentage
Mean age (years) (standard deviation)	43.3 (8.7) years	
*Gender*		
Male	67	69.8%
*Where did you complete your Nephrology fellowship training?*		
Saudi board	31	32.3%
Masters or PhD/fellowship in Egypt	23	24.0%
Royal college of physician and surgeons of Canada/fellowship in Canada	15	15.6%
American board of internal medicine (nephrology)/fellowship in USA	6	6.3%
Royal colleges of physicians of the UK (MRCP)/fellowship in UK	5	5.2%
Arab board	1	1.0%
Others	15	15.6%
*Mean number of years you have been practicing nephrology* (standard deviation)	12.3 (9.2) years	
*What is your primary practice setting?* (You can choose more than 1)		
Ministry of health hospital (MOH)	42	43.8%
Non-Ministry of health hospital	29	30.2%
Private hospital	18	18.8%
Outpatient dialysis units only	14	14.6%
*Region of nephrology practice*		
Central region	11	11.5%
Western region	65	67.7%
Northern region	1	1.0%
Eastern region	15	15.6%
Southern region	4	4.2%

### 3.1. Practice settings exercise and PA characteristics

The majority of the population of the nephrology practice is adults (93.8%) and only a very small proportion is pediatrics (6.3%). In relation to exercise, 10.4% of the respondents exercise almost never. Only 9.4% of renal units have exercise programs available to patients. These programs are available mainly for advanced CKD (pre-dialysis) and in-center hemodialysis patients, where 66.7% each of the units have them, and 33.3% have them for home therapy and renal transplant patients. The major barriers for initiating or expanding exercise programs at their centers are a lack of motivation/interest from front-line staff (55.2%), lack of interest from management (48.3%), and no funding (47.1) (Table 2).

**Table 2:** Practice settings exercise and physical activity characteristics.

Variable	Frequency	Percentage
*The age of practice population*		
Adults	90	93.8%
Pediatrics	6	6.3%
*How often do you exercise or do physical activity in your leisure time?*		
Almost never	10	10.4%
Less than once a week	22	22.9%
Once a week	18	18.8%
2–3 times a week	28	29.2%
4–5 times a week	9	9.4%
Daily	9	9.4%
*Does your renal unit offer exercise program to patients?*		
Yes	9	9.4%
*Which patient groups at your facility have access to exercise resources provided by your renal program?* (select all that apply) (n = 9)		
Advanced CKD (pre-dialysis) patients	6	66.7%
In-center hemodialysis patients	6	66.7%
Home therapies (peritoneal dialysis or home dialysis)	3	33.3%
Renal transplant patients	3	33.3%
*In your opinion, what barriers exist at your center to implementing or expanding an exercise program? (select all that apply) (n = 87*)		
Lack of motivation/interest in such programs from the front-line staff	48	55.2%
Lack of interest from management/people who run the program	42	48.3%
No funding	41	47.1%
Patients would not exercise or are not interested	31	35.6%
Difficulty with transportation (getting participants to the exercise program)	22	25.3%
Concerns for patient safety	18	20.7%

### 3.2. Healthcare providers’ practices and opinions on physical activity for CKD patients

In their practice, 56.2% of respondents (22.9% always and 33.3% frequently) ask patients about their level of PA. Additionally, 64.6% (32.3% always and 32.3% frequently) give patients specific advice on how to increase their level of PA. Regarding the adequacy of evidence supporting regular exercise for CKD patients, 26.0% of respondents believe there is not enough evidence, while 34.4% are uncertain. For the types of exercise most beneficial for people with CKD, 90.6% recommend walking, 35.4% frequently recommend cycling, and 33.3% recommend strengthening exercises. Finally, 34.4% of respondents believe that physiotherapists should take ownership in providing exercise counseling and resources to people with CKD (Table 3).

**Table 3:** Healthcare providers’ practices and opinions on physical activity for CKD patients.

Variable	Frequency	Percentage
*In your practice, how often do you ask patients about their level of physical activity?*		
Always	22	22.9%
Frequently	32	33.3%
Occasionally	29	30.2%
Infrequently	10	10.4%
Never	3	3.1%
*In your practice, do you give patients specific advice on how to increase their level of physical activity?*		
Always	31	32.3%
Frequently	31	32.3%
Occasionally	25	26.0%
Infrequently	7	7.3%
Never	2	2.1%
*In your opinion, is there enough evidence in the literature to support regular exercise* *prescription and participation by all patients with CKD (all stages)?*		
Yes	38	39.6%
No	25	26.0%
Don’t know	33	34.4%
*In your opinion, is there any type of exercise that would be most beneficial for people* *with CKD to participate in?*		
Walking	87	90.6%
Cycling	34	35.4%
Strengthening	32	33.3%
Flexibility	24	25.0%
None would be beneficial	1	1.0%
Others	1	1.0%
*In your opinion, who should take ownership in providing exercise counseling and resources to people with CKD?*		
Physiotherapist	33	34.4%
Nephrologist	31	32.3%
Renal allied health professional (nurse, dietician)	16	16.7%
Primary care physician	10	10.4%
All of the above	4	4.2%
None of the above	2	2.1%

### 3.3. Healthcare providers’ confidence, concerns, and perceptions about exercise for CKD patients

The majority agreed or strongly agreed to recommend exercise in patients with CKD (76.0%). Concerns or uncertainties about safety of exercise in this population were reported in 32.3%. Fifty percent agreed or strongly agreed that people with CKD are as likely to exercise if advised as people without CKD. Exercise benefits are viewed positively: 96.9% believed that regular exercise is beneficial to the general population, 92.7% to those with mild-moderate CKD, 79.2% to advanced CKD patients, and 78.1% to those on dialysis. Furthermore, 74.0% thought exercise might reduce symptoms associated with dialysis, such as restless legs or cramping (Table 4).

**Table 4:** Healthcare providers’ confidence, concerns, and perceptions about exercise for CKD patients.

Variable	Strongly agree	Agree	Neutral	Disagree	Strongly disagree
I am confident in my ability to recommend exercise to people with CKD.	35.4%	40.6%	22.9%	1.0%	0.0%
I am concerned or unsure regarding the safety of exercise in people with CKD.	11.5%	20.8%	27.1%	35.4%	5.2%
If advised to do so, people with CKD are as likely to exercise as people without CKD.	17.7%	32.3%	17.7%	31.3%	1.0%
For people in the general population (no CKD), regular exercise has health benefits.	80.2%	16.7%	3.1%	0.0%	0.0%
For people with mild-moderate CKD, regular exercise has health benefits.	66.7%	26.0%	5.2%	2.1%	0.0%
For people with advanced CKD, regular exercise has health benefits.	39.6%	39.6%	15.6%	4.2%	1.0%
For people on dialysis, regular exercise has health benefits.	38.5%	39.6%	19.8%	1.0%	1.0%
Exercise can help with dialysis-related symptoms (e.g., restless legs, cramping)	32.3%	41.7%	22.9%	3.1%	0.0%

### 3.4. Physicians’ attitude towards exercise for CKD patients

The mean attitude score for the study participants was 32.1 (SD: 3.9) out of 40 (equal to 80.3%); which demonstrates positive attitude towards recommending exercise for CKD patients. Binary logistic regression analysis identified that there is no statistically significant difference between physicians in term of their attitude towards recommending exercise for CKD patients (*P* > .05) (Table 5).

**Table 5:** Physicians’ attitude towards exercise for CKD patients.

Variable	Odds of having positive attitude	*P*-value
*Mean age*		
Less than or equal to 43.3 years	1.00	
Above 43.3 years	1.26 (0.56–2.83)	.576
*Gender*		
Males	1.00	
Females	0.80 (0.34–1.92)	.623
*Where did you complete your Nephrology fellowship training?*		
Saudi board	1.00	
Masters or PhD/fellowship in Egypt	0.13 (0.01–1.29)	.081
Royal college of physician and surgeons of Canada/fellowship in Canada	0.40 (0.04–4.41)	.454
American board of internal medicine (nephrology)/fellowship in USA	0.13 (0.01–2.18)	.158
Royal colleges of physicians of the UK (MRCP)/fellowship in UK	0.14 (0.02–1.39)	.094
Arab board	0.40 (0.02–10.02)	.577
Others	0.40 (0.02–10.02)	.577
*Years you have been practicing nephrology*		
Less than or equal to 12.3 years	1.00	
Above 12.3 years	0.90 (0.40–2.02)	.804
*What is your primary practice setting?*		
Ministry of health hospital (MOH)	1.00	
Non-Ministry of health hospital	0.82 (0.28–2.38)	.818
Private hospital	1.00 (0.28–3.61)	1.00
Outpatient dialysis units only	0.83 (0.22–3.16)	.833
*Region of nephrology practice:*		
Central region	1.00	
Western region	1.75 (0.17–17.69)	.635
Northern region	0.86 (0.11–6.46)	.881
Eastern region	–	
Southern region	0.67 (0.07–6.11)	.720

## 4. Discussion

This study provides a comprehensive analysis of current practices, attitudes, and obstacles regarding exercise and PA for patients with CKD among nephrologists in Saudi Arabia. One of the important findings was that only 9.4% of renal units have exercise programs available to patients, which is consistent with the results of other studies. For instance, a previous study revealed that only 26% of Italian nephrology centers offer exercise programs.^[27]^ A prior international study found that a substantial percentage of centers in Australia, New Zealand, and Canada lack exercise programs for patients with CKD.^[19]^ In Australian renal units, the education programs involved only 9% of their scope on exercise practice.^[28]^ Earlier studies also found that less than 1 in ten dialysis units globally provide such programs except for some countries.^[29–31]^ Likewise, only 19.3% of nephrology services in Spain have exercise programs for CKD patients.^[32]^ These findings highlight the widespread concern about the lack of utilized exercise interventions despite their well-documented advantages for CKD patients. Thus, it underlines the demand for enhanced approaches to integrate exercise programs into renal units.

In this study, we found that exercise and PA programs are available mainly for advanced CKD (pre-dialysis) and in-center hemodialysis patients, where 66.7% of each renal unit have them, and 33.3% have them for home therapy and renal transplant patients. Consistent with our findings, earlier research has revealed that exercise programs focus on individuals with more advanced CKD and those undergoing hemodialysis. In Italy, exercise interventions target mostly hemodialysis patients (69%), with less availability for patients among the first 3 CKD stages (19%).^[27]^ Likewise, exercise programs are offered primarily to hemodialysis patients in Spain.^[32]^ However, the study conducted in Italy indicates that exercise interventions are also provided substantially to renal transplant patients (56%).^[27]^ All these findings suggest that patients with advanced CKD and those undergoing hemodialysis require exercise interventions more significantly as compared to other CKD patient groups. Studies indicate a high prevalence of sarcopenia in CKD patients, particularly in the advanced stages of the condition.^[33–35]^ In the case of dialysis patients, there is also a tendency to develop severe sarcopenia and low muscle strength.^[36]^ Besides, patients with advanced CKD and those undergoing dialysis have a raised cardiovascular complications risk.^[37–41]^ Regular exercise can reduce these risks and improve general patient well-being.^[42]^

Our findings regarding the limited exercise programs for home therapy and renal transplant patients may arise out of many challenges. First, according to previous research, nephrologists may not generally consider prescribing home exercise programs as a part of their responsibilities.^[43]^ This may result in a gap in care for home therapy CKD patients whose contact with health care providers is limited. Besides, transplant patients need special exercise programs that should consider their complicated medical conditions.^[44]^ The limited availability of such programs emphasizes the necessity of specialized exercise programs that consider each stage of CKD and each treatment strategy.

In this study, the principal barriers to initiating or expanding exercise programs at their centers are a lack of motivation/interest from front-line staff (55.2%), lack of interest from management (48.3%), and no funding (47.1%). These findings align with barriers reported in several prior studies,^[18–24,45–48]^ suggesting common challenges to promoting PA in the CKD patient population. For example, a study in nephrology centers in Italy showed that the main reported barriers were a deficiency of resources and institutional support for physical exercise (46%).^[27]^ In Nigeria, members of the renal care team also identified several related barriers, including an unmotivated patient, 75%; limited resources, 69%; insufficient funds, 66%; and an unmotivated staff, 63%.^[49]^ Overcoming these barriers requires additional institutional support, exercise program funding, further training and resources for health staff, and educating the patient about the importance of PA and options for exercise.

In their practice, 56.2% of respondents (22.9% always and 33.3% frequently) in this study asked patients about their level of PA. Besides, around 64.6% (32.3% always and 32.3% frequently) give patients specific advice on how to increase their level of PA. These results align with previous studies. A prior study found that the United Kingdom (UK) renal multidisciplinary teams frequently inquire about patients’ PA; in particular, 74% of participants inquire about PA, with 59% of them also providing consultation and 42% recommending PA.^[18]^ In an international study, 71% of nephrologists documented always or frequently inquiring about patients’ PA levels, and 47% stated that they always or frequently provide specific exercise advice.^[19]^ While acknowledging the benefits of counseling on exercise, there are other barriers to overcome; for example, nephrologists often face competing priorities expected to limit the frequency of patient consultations concerning their PA.^[19]^

In this study, regarding the adequacy of evidence supporting regular exercise for CKD patients, 26.0% of respondents believe there is insufficient evidence, while 34.4% are uncertain. These underscore a considerable lack of awareness among nephrologists regarding the benefits of exercise. A prior international survey has shown only 38% of nephrologists acknowledged the sufficiency of evidence to recommend exercise, and 35% admitted unfamiliarity with the relevant literature.^[19]^ Despite abundant and compelling evidence supporting the positive impact of PA on CKD patients,^[10,45,50]^ a significant number of healthcare professionals seem to be uninformed or unsure about the robustness of this evidence. Contrary to this, a prior study revealed that 93% of nephrology centers in Italy assume that there is substantial scientific evidence endorsing the advantages of periodic exercise programs for patients with CKD.^[27]^ Another study by Anding-Rost et al found that exercises during hemodialysis for patients with chronic kidney failure improved the functional mobility and physical performance.^[51]^

Our findings regarding the most beneficial exercises for CKD patients reveal that 90.6% of our respondents recommend walking, 35.4% frequently recommend cycling, and 33.3% recommend strengthening exercises. These align with earlier studies that underscore the significance of different types of PA for individuals with chronic kidney disease. According to a previous review, the prevalent exercise types of CKD patients are aerobic exercise, including cycling and walking.^[45]^ Multiple Italian nephrology centers believe aerobic exercise is highly beneficial for patients with CKD, with 81% of them considering it the most advantageous type of exercise, followed by 12% of centers choosing muscle strengthening.^[27]^ Besides, programs including combined aerobic and resistance exercises are the most common programs prescribed by nephrology services in Spain.^[32]^

An earlier systematic review and meta-analysis found that aerobic capacity significantly increased in patients with CKD at stages II-V and in the transplantation and hemodialysis groups,^[13]^ reflecting the broad benefits of aerobic exercise. Besides, a prior study observed that aerobic training exercises improve health-related quality of life, high-density lipoprotein cholesterol level, exercise duration, and cardio-pulmonary function in adult CKD patients.^[52]^

Resistance training, although less frequently recommended, is essential for preventing and addressing CKD that is induced by muscle atrophy.^[53]^ Moreover, research provides evidence that supports the effectiveness of resistance training in enhancing cardiorespiratory function, inflammatory response, metabolic disorder, and physical function.^[54]^ These emphasize the significance of integrating muscle-strengthening exercises into the care of CKD patients.

In our study, around 34.4% of respondents believe that physiotherapists should take ownership in providing exercise counseling and resources to people with CKD. In line with this, in Italy, 73% of nephrologists believe that physiotherapists or exercise physiologists should be responsible for prescribing exercise.^[27]^ Besides, a multinational study revealed that 71% of nephrologists assumed that providing exercise counseling and resources for CKD patients was the role of the physiotherapist, exercise physiologist, or kinesiologist.^[19]^ In addition, in Spain, “professionals in the sciences of PA and sport” and physiotherapists prescribe approximately 75% of the physical exercise programs for CKD patients.^[32]^ These highlight the international recognition of the importance of involving various healthcare professionals and emphasize the need for collaborative efforts to incorporate PA into CKD management.

In our study, the mean attitude score for the study participants was 32.1 (SD: 3.9) out of 40 (equal to 80.3%), which demonstrates a positive attitude toward recommending exercise for CKD patients. These were additionally reinforced by positive beliefs in the benefits resulting from regular exercise, with 96.9% of precipitants believing that regular exercise is beneficial to the general population, 92.7% to those with mild-moderate CKD, 79.2% to advanced CKD patients, and 78.1% to those on dialysis. These findings are consistent with available evidence that health professionals recognize the significant benefits of PA at all stages of CKD,^[19]^ whereby exercises have an imperative role in improving patient outcomes and quality of life.^[8,12,13]^

The Italian Society of Nephrology has verified that CKD patients receive a variety of benefits from regular PA and exercise training, such as an overall enhancement in quality of life, improved physical function, and enhanced cardiometabolic and neuromuscular function.^[55]^ Additionally, exercise has the potential to reduce mortality and provide nephroprotection. These advantages are consistent across all phases of CKD, including those who are undergoing conservative therapy, kidney replacement therapy (hemodialysis or peritoneal dialysis), and kidney transplant recipients. Additionally, PA and exercise training are secure for CKD patients when they are implemented with the requisite precautions.^[55]^ Customized exercise programs and gradual PA should be implemented in accordance with the patient’s exercise tolerance, which has the potential to improve compliance. Clinicians are advised to implement a sequence of questionnaires and assessments to evaluate the patient’s level of PA and performance. Nevertheless, the implementation of exercise and PA in clinical practice is impeded by numerous obstacles that are associated with patients and healthcare professionals. The proactive role of nephrologists is essential in overcoming these barriers. They should actively integrate exercise training and promote PA into routine care plans.^[55]^ For the comprehensive rehabilitation of CKD patients, it is essential to employ a multidisciplinary team approach that includes nephrologists, nurses, exercise professionals, and dietitians. The efficacy of these interventions could be further improved by incorporating remote checkups and novel technologies.^[55]^

This study has limitations. The use of professional networks, email invitations, and various social media channels to maximize the reach and participation make it hard estimate the response rate for this study, which might lead to nonresponse bias (selection bias). Therefore, our findings should be interpreted carefully.

## 5. Conclusion

This study highlights a critical gap in the availability of exercise programs for CKD patients. Key barriers include lack of interest by staff and management and funding issues. In spite of these barriers, a majority of physicians acknowledge the role of exercise in CKD patients and advice regarding PA on a frequent basis. It suggests recommendations in order to expand the programs, including increasing staff motivation, securing management support, and getting funding for exercise programs and identifying the role of physiotherapists in exercise counseling for CKD patients.

## Acknowledgments

We would like to thank the Saudi Society of Nephrology for their support in conducting this study.

## Author contributions

**Conceptualization:** Enad Alsolami, Sami Alobaidi.

**Data curation:** Enad Alsolami, Sami Alobaidi.

**Formal analysis:** Enad Alsolami, Sami Alobaidi.

**Funding acquisition:** Enad Alsolami, Sami Alobaidi.

**Investigation:** Enad Alsolami, Sami Alobaidi.

**Methodology:** Enad Alsolami, Sami Alobaidi.

**Project administration:** Enad Alsolami.

**Resources:** Enad Alsolami, Sami Alobaidi.

**Software:** Enad Alsolami, Sami Alobaidi.

**Supervision:** Enad Alsolami.

**Validation:** Enad Alsolami, Sami Alobaidi.

**Visualization:** Enad Alsolami, Sami Alobaidi.

**Writing – original draft:** Enad Alsolami, Sami Alobaidi.

**Writing – review & editing:** Enad Alsolami, Sami Alobaidi.
